# Supplementary material for: Identification of four biotypes in temporal lobe epilepsy via machine learning on brain images
Source: Nat Commun. 2024 Mar 12;15:2221. doi: 10.1038/s41467-024-46629-6 (PMC10933450; doi:10.1038/s41467-024-46629-6)
Supplement: Supplementary file 1 — Supplementary Information [file 41467_2024_46629_MOESM1_ESM.pdf]

Supplementary Material

Identification of four biotypes in temporal lobe epilepsy via machine learning on brain images

Jiang et al.

This supplementary material includes as follows:

Supplementary Methods

Method S1. Total intracranial volume (TIV) analyses.

Method S2. Permutation Test.

Method S3. MRI Follow-up Analysis.

Method S4. Disease Duration Subsample Analysis.

Method S5. Hippocampal Sclerosis Analysis.

Supplementary Tables

Supplementary Table 1. ROI-wise correlation coefficients between SuStaIn stages and regional z scores.

Supplementary Table 2. Regional z score for each subtype.

Supplementary Table 3. T values of ROI-wise z score comparisons between any two subtypes in addition to between subtypes and healthy controls.

Supplementary Table 4. Sequence of biomarkers for each trajectory via SuStaIn.

Supplementary Table 5. Consistency of individual subtype label for SuStaIn model based on 23 ROI features and 13 ROI features.

Supplementary Table 6. Consistency of individual subtype label for SuStaIn model generalized to unseen data and original subtype.

Supplementary Table 7. Prediction performance of classifiers on the test data.

Supplementary Table 8. Regions of interest (ROIs) included for SuStaIn modeling.

Supplementary Table 9. Results of linear/logistic regress model with HS as an independent variable.

Supplementary Table 10. Results of linear/logistic regress model with SuStaIn subtype as an independent variable.

Supplementary Figures

Supplementary Figure 1. ROI-wise z score of bilateral amygdala volume.

Supplementary Figure 2. Comparisons of ROI-wise z score between any two subtypes in addition to between subtypes and healthy control group (HC).

Supplementary Figure 3. Four distinct neuroanatomical signatures of brain atrophy patterning in people with temporal lobe epilepsy in discovery dataset and validation dataset separately.

Supplementary Figure 4. Machine learning prediction procedures through a framework under SuStaIn subtype.

Supplementary Figure 5. Prediction performance of Machine learning classifier on identifying the subject who achieves seizure freedom or not after surgery.

Supplementary Figure 6. Null hypothesis distributions are shown for prediction performance (Younden Index J).

Supplementary Figure 7. Optimal number of clusters.

Supplementary Figure 8. Positional variance diagrams.
Supplementary Figure 9. Probability of maximum likelihood trajectory is high across almost all SuStaIn stages.
Supplementary Figure 10. A flowchart of machine learning procedures to predict treatment outcome.
Supplementary Figure 11. Subtype labels remain consistent for almost all patients at baseline and follow-up.
Supplementary Figure 12. Spatiotemporal trajectories of brain atrophy in short-term and long-term subsamples.
Supplementary Figure 13. Consistency of individual staging between stages of unseen data and original result.
Supplementary Figure 14. Comparisons of total intracranial volume (TIV) among subtype 1, 2, 3, 4 and healthy control (HC).

Method S1. Total intracranial volume (TIV) analyses

We found a subtype effect on total intracranial volume (TIV) — individuals with the cortical subtype (subtype 3) apparently had significantly larger TIV than the other three subtypes. To clarify it, we conducted the following analyses.

1) We compared the TIV difference between subtypes and healthy control group (HC) by ANOVA; post-hoc comparisons (Bonferroni method) were also conducted to compare difference between any of two subgroups. We found that compared with HC, the TIV was significantly smaller in subtype 1 ($p<0.001$), subtype 2 ($p=0.004$) and subtype 4 ($p<0.001$), but larger in subtype 3 ($p=0.036$). This comparison is shown in the **Supplementary Figure 14**.

2) To examine whether TIV was associated with specific clinical features, we investigated the correlation between TIV and age of onset as well as illness duration using Pearson correlation analysis. We found there was no significant correlation between TIV and age of onset ($r=0.028$, $p=0.632$) or illness duration ($r=-0.107$, $p=0.066$) in the patient group. In addition, we compared the difference of TIV between clinical subgroups using ANOVA. We found that the TIV was significantly smaller in the HS subgroup compared to the non-HS subgroup ($F=7.28$, $p=0.007$). The FS seizure type subgroup showed smaller TIV than the BECT seizure type subgroup ($F=4.03$, $p=0.045$). In addition, the male patient group showed larger TIV than the female patient group ($F=53.5$, $p<0.001$). Besides, there was no significant difference of TIV between clinical subgroups ($p>0.05$) in term of other clinical features, including seizure lateralization, history of hypoxia at birth, history of head trauma, history of febrile seizures, history of encephalitis meningitis, history of positive family, aura, seizure frequency, seizure type, medications, pathology waves and treatment outcomes. These results indicated that TIV may not drive the differences of clinical features between-subtypes, although the subtype 3 showed a larger TIV than other subtypes.

Method S2. Permutation Test

To examine whether the prediction performance is significantly better than random predictions, we used a permutation test to evaluate significance by random permutation of predictive label. Specifically, the label (seizure-free or not) of subject were randomly permuted across all subjects. Subsequently, we re-conducted machine learning procedures (**Supplementary Figure 10**), and predicted the label of each subject on test set using leave-one subject-out cross-validation (LOOCV). Prediction performance was evaluated using Youden Index. The above random permutation was repeated 1000 times, yielding a distribution of random prediction performance. The significance of true prediction performance was estimated by its location within the distribution of random prediction performance. One-side $P<0.05$ (i.e., better than 95% of random predictions) was considered as a significance that rejects the null hypothesis.

Method S3. MRI Follow-up Analysis

We follow up brain MRI data of the part of individuals with TLE (n=23, without surgery until follow-up). The average of interval time between the baseline scanning and follow-up scanning is 39.0 months (SD=16.8 months), range from 10.5 to 76.7 months. Using this subsample, we re-estimated the SuStaIn subtype labels of these individuals using their follow-up MRI data. We examined whether the subtype label at follow-up keeps consistent with the baseline label. We found that subtype labels remained consistent for almost all patients at baseline and follow-up (**Supplementary Figure 11**), except for two patients with subtype 4 (i.e., stage=0) who converted to subtype 1 and 3 at follow-up, respectively. This result suggests that once certain initial brain injury is established, it is less likely to shift from one trajectory pattern (i.e., subtype) to another. This assumption is also supported by **Supplementary Figure 9**, which shows that the probability of maximum likelihood subtype is high across all SuStaIn stage, indicating that there was no "cross-over events" in the subtype sequence.

Method S4. Disease Duration Subsample Analysis

We divided all of individuals with TLE (n=296) into two disease subgroup according to their disease durations (cutoff = median value (i.e., 9.5 years)), yielding a short-term subgroup (n=148, mean disease duration=4.8±2.7 years) and a long-term subgroup (n=148, mean disease duration=17.5±7.4 years). Such a subgrouping rule take into account the same size of subsamples. We re-estimated the ‘spatiotemporal patterns of brain atrophy’ (i.e., SuStaIn trajectory) in each subgroup, separately. We found there was a similar pattern of the three trajectories (left hippocampus-led, right hippocampus-led and cortex-led) in the two disease subgroups (**Supplementary Figure 12**). This result suggests that the distinct spatiotemporal patterns of brain atrophy may not be affected by disease progress.

Method S5. Hippocampal Sclerosis Analysis

In our data, we examined the associations between HS and other clinical variables (age of onset, illness duration, medication outcome and surgery outcome) using a linear regress or logistic model analysis. We observed that patients with HS+ show younger age of onset compared to those HS- patients (t=-3.49, p=0.001). In addition, we found that patients with HS- experience worse surgical outcomes compared to those HS+ patients (χ^2=5.99, p=0.014) (**see Supplementary Table 9**). To examine whether the clinical differences among SuStaIn subtypes are affected by HS, we re-analyzed the correlations between clinical features and subtype with HS effect as a covariate using a linear regress or logistic regress model ($Y \sim X+C+\varepsilon$). Here, clinical variable is dependent variable (*Y*); SuStaIn subtype is independent variable (*X*); and HS is covariate variable (*C*). After controlling HS effect, we still found significant associations of SuStaIn subtype with age of onset, illness duration and medication outcomes (**see Supplementary Table 10**). This suggests that the clinical differences between SuStaIn subtypes remain significant after controlling HS effect.

Supplementary Table 1. ROI-wise correlation coefficients between SuStaIn stages and regional z scores.

Features	Trajectory 1 (n=85)		Trajectory 2 (n=113)		Trajectory 3 (n=41)	
	r	p	r	p	r	p
Mean_of_cortical_regions	.599**	0.000	.791**	0.000	.847**	0.000
L_thalamus	.659**	0.000	.598**	0.000	.404*	0.009
L_putamen	.419**	0.000	.537**	0.000	-0.022	0.890
L_hippocampus	.627**	0.000	.577**	0.000	.431*	0.005
R_thalamus	.452**	0.000	.426**	0.000	0.17	0.287
R_pallidum	.373**	0.000	.513**	0.000	0.256	0.106
R_hippocampus	.269*	0.013	0.157	0.097	-0.006	0.973
R_caudalmiddlefrontal	.446**	0.000	.745**	0.000	.598**	0.000
R_paracentral	.464**	0.000	.656**	0.000	.685**	0.000
R_parsopercularis	.462**	0.000	.621**	0.000	.367*	0.018
R_parstriangularis	.417**	0.000	.611**	0.000	.540**	0.000
R_precentral	.491**	0.000	.686**	0.000	.684**	0.000
R_precuneus	.415**	0.000	.729**	0.000	.810**	0.000
R_superiorfrontal	.456**	0.000	.679**	0.000	.759**	0.000
L_caudalmiddlefrontal	.455**	0.000	.710**	0.000	.721**	0.000
L_entorhinal	.499**	0.000	.458**	0.000	.545**	0.000
L_fusiform	.580**	0.000	.664**	0.000	.687**	0.000
L_parahippocampal	.485**	0.000	.462**	0.000	.692**	0.000
L_paracentral	.393**	0.000	.713**	0.000	.515**	0.001
L_precentral	.517**	0.000	.689**	0.000	.753**	0.000
L_precuneus	.590**	0.000	.675**	0.000	.787**	0.000
L_superiorfrontal	.507**	0.000	.683**	0.000	.558**	0.000
L_temporalpole	.458**	0.000	.425**	0.000	.406**	0.009
L_transversetemporal	.236*	0.029	.385**	0.000	.657**	0.000

**p<0.001, *p<0.05, two-sided. Spearman correlation test is conducted for data analysis. Multiple comparisons were corrected by FDR.

Supplementary Table 2. Regional z score for each subtype.

Region	Subtype1		Subtype2		Subtype3		Subtype4	
	mean	sd	mean	sd	mean	sd	mean	sd
Right_bankssts	-0.424	1.061	0.098	1.302	0.921	0.861	-0.659	1.033
Right_caudalanteriorcingulate	-0.477	1.024	-0.511	1.189	0.553	0.701	-0.837	1.032
Right_caudalmiddlefrontal	-0.386	1.096	0.091	1.162	1.453	0.848	-0.814	0.977
Right_cuneus	-0.638	0.975	-0.192	1.124	0.707	0.911	-0.761	1.047
Right_entorhinal	0.148	1.232	0.744	1.287	0.874	1.274	-0.235	1.196
Right_fusiform	-0.721	0.924	-0.220	1.250	0.968	0.770	-0.999	0.910
Right_inferiorparietal	-0.503	0.890	0.062	1.128	1.210	0.735	-1.025	1.079
Right_inferiortemporal	-0.513	0.810	0.029	1.017	1.033	0.707	-0.647	0.912
Right_isthmuscingulate	-0.371	0.830	-0.130	1.066	0.711	0.759	-0.662	0.873
Right_lateraloccipital	-0.448	0.884	-0.034	1.163	0.985	0.745	-0.947	1.033
Right_lateralorbitofrontal	-0.422	0.906	-0.346	1.082	0.857	0.863	-0.770	0.980
Right_lingual	-0.544	0.768	-0.179	1.130	0.760	0.819	-0.698	0.976
Right_medialorbitofrontal	-0.507	0.869	-0.329	1.069	0.758	0.649	-0.747	0.819
Right_middletemporal	-0.554	0.892	0.115	1.155	0.966	0.777	-0.736	0.989
Right_parahippocampal	-0.504	1.137	-0.152	1.195	0.506	1.159	-0.587	1.112
Right_paracentral	-0.362	0.859	0.080	1.053	1.088	0.680	-0.903	0.892
Right_parsopercularis	-0.345	0.929	-0.096	1.057	1.095	0.658	-0.674	0.878
Right_parsorbitalis	-0.198	0.974	-0.044	1.068	1.264	0.833	-0.406	1.046
Right_parstriangularis	-0.279	0.910	-0.105	1.109	1.255	0.645	-0.730	0.923
Right_pericalcarine	-0.705	1.010	-0.496	1.004	0.638	0.638	-0.982	1.213
Right_postcentral	-0.469	1.010	-0.083	1.208	1.072	0.886	-0.907	0.970
Right_posteriorcingulate	-0.533	0.865	-0.279	1.124	0.864	0.794	-0.872	1.037
Right_precentral	-0.253	0.892	0.129	1.077	1.139	0.608	-0.685	0.763
Right_precuneus	-0.598	1.077	0.016	1.215	1.231	0.852	-1.036	0.980
Right_rostralanteriorcingulate	-0.549	0.884	-0.483	1.170	0.863	0.743	-0.756	0.967
Right_rostralmiddlefrontal	-0.498	0.921	-0.264	1.042	1.127	0.564	-0.811	1.029
Right_superiorfrontal	-0.598	1.015	-0.149	1.070	1.279	0.574	-0.903	0.956
Right_superiorparietal	-0.515	1.059	0.072	1.235	1.166	0.765	-1.067	1.101
Right_superiortemporal	-0.584	1.034	0.129	1.160	1.028	0.896	-0.981	0.874
Right_supramarginal	-0.441	0.931	0.073	1.139	1.096	0.790	-0.875	0.948
Right_frontalpole	-0.541	0.851	-0.339	1.157	0.913	0.874	-0.619	0.870
Right_temporalpole	-0.194	0.969	0.403	1.016	0.712	0.755	-0.574	1.075
Right_transversetemporal	-0.264	0.967	-0.071	0.976	0.751	0.702	-0.370	0.919
Right_insula	-0.623	0.953	-0.135	1.183	0.962	0.844	-0.827	1.030
Left_bankssts	-0.160	1.004	-0.197	1.074	0.890	0.853	-0.905	0.964
Left_caudalanteriorcingulate	-0.436	0.988	-0.456	1.154	0.536	0.864	-0.752	0.912
Left_caudalmiddlefrontal	-0.181	0.968	0.007	1.162	1.332	0.699	-0.772	0.940
Left_cuneus	-0.509	1.009	-0.317	1.008	0.680	0.893	-0.805	1.007
Left_entorhinal	0.300	1.118	0.061	0.993	0.621	1.127	-0.213	1.073
Left_fusiform	-0.227	1.055	-0.308	1.229	0.995	0.821	-0.836	0.865
Left_inferiorparietal	-0.248	0.970	-0.110	1.302	1.221	0.772	-1.017	1.137

Left_inferiortemporal	-0.319	0.785	-0.359	1.145	0.861	0.698	-0.778	1.008
Left_isthmuscingulate	-0.462	0.941	-0.324	1.099	0.701	0.704	-0.622	0.890
Left_lateraloccipital	-0.394	0.936	-0.226	1.266	0.977	0.876	-0.914	0.969
Left_lateralorbitofrontal	-0.352	0.792	-0.352	1.074	0.845	0.741	-0.667	0.969
Left_lingual	-0.527	0.940	-0.484	1.201	0.519	0.839	-0.803	0.997
Left_medialorbitofrontal	-0.388	0.855	-0.309	1.182	0.960	0.679	-0.731	0.944
Left_middletemporal	-0.133	0.983	-0.161	1.121	1.083	0.782	-0.748	0.888
Left_parahippocampal	-0.206	1.199	-0.139	1.114	0.569	1.168	-0.513	1.130
Left_paracentral	-0.012	1.055	0.119	1.139	1.206	0.763	-0.719	0.843
Left_parsopercularis	-0.323	0.936	-0.092	1.145	1.211	0.744	-0.674	0.794
Left_parsorbitalis	-0.317	0.969	-0.302	1.180	1.159	0.820	-0.801	1.148
Left_parstriangularis	-0.313	0.860	-0.193	1.078	1.034	0.718	-0.834	1.060
Left_pericalcarine	-0.789	1.012	-0.661	1.141	0.615	1.019	-0.966	1.292
Left_postcentral	-0.344	0.955	-0.161	1.098	1.065	0.797	-0.877	1.000
Left_posteriorcingulate	-0.492	0.840	-0.416	1.206	0.815	0.709	-0.784	0.885
Left_precentral	-0.196	1.021	0.069	1.081	1.240	0.685	-0.726	0.888
Left_precuneus	-0.149	0.978	-0.001	1.052	1.119	0.778	-0.794	0.911
Left_rostralanteriorcingulate	-0.510	1.038	-0.320	1.011	0.979	0.843	-0.806	1.009
Left_rostralmiddlefrontal	-0.411	0.864	-0.314	1.034	1.077	0.586	-0.784	0.901
Left_superiorfrontal	-0.395	0.964	-0.234	1.093	1.243	0.551	-0.844	0.907
Left_superiorparietal	-0.140	1.088	0.002	1.197	1.153	0.855	-1.024	1.158
Left_superiortemporal	-0.024	1.205	-0.184	1.259	1.158	0.859	-0.974	0.919
Left_supramarginal	-0.166	1.007	0.003	1.180	1.425	0.808	-0.898	1.022
Left_frontalpole	-0.337	0.786	-0.206	1.156	0.895	0.793	-0.609	1.134
Left_temporalpole	0.057	1.038	0.087	1.046	0.826	0.977	-0.539	1.105
Left_transversetemporal	-0.319	1.084	-0.143	1.007	0.809	1.015	-0.977	1.166
Left_insula	-0.577	0.980	-0.328	1.030	0.688	0.878	-0.867	0.965
Left_Accumbens_area	-0.225	1.147	-0.233	1.179	-0.526	1.268	-0.677	1.064
Left_Amygdala	0.588	1.502	-0.327	1.333	-0.694	1.271	-1.446	1.419
Left_Caudate	0.202	1.011	-0.016	1.071	-0.007	0.947	-0.064	0.844
Left_Hippocampus	4.093	1.385	0.609	1.521	0.161	1.824	-0.767	1.377
Left_Pallidum	0.382	0.897	0.234	1.149	0.090	0.980	-0.161	0.885
Left_Putamen	0.138	1.010	-0.029	1.060	-0.155	0.989	-0.406	0.948
Left_Thalamus	1.649	1.555	0.697	1.149	0.335	0.999	-0.079	0.954
Right_Accumbens_area	-0.369	0.947	-0.039	1.164	-0.530	0.950	-0.713	1.040
Right_Amygdala	-0.451	0.987	0.539	1.006	-0.640	1.019	-1.325	1.340
Right_Caudate	-0.154	0.896	0.043	1.019	0.020	0.982	-0.241	0.840
Right_Hippocampus	-0.189	1.105	4.058	1.234	0.021	1.150	-0.718	1.419
Right_Pallidum	0.285	0.801	0.308	1.031	0.206	0.936	-0.322	0.958
Right_Putamen	-0.081	1.112	0.308	1.194	-0.247	0.994	-0.562	0.884
Right_Thalamus	0.563	1.169	1.722	1.245	0.534	0.840	0.097	0.840

Supplementary Table 3. T values of ROI-wise z score comparisons between any two subtypes in addition to between subtypes and healthy controls.

Region	S1 vs. S2	S1 vs. S3	S1 vs. S4	S2 vs. S3	S2 vs. S4	S3 vs. S4	S1 vs. HC	S2 vs. HC	S3 vs. HC	S4 vs. HC
Right_bankssts	-3.002	-7.014	1.297	-3.735	3.799	7.913	-3.664	0.795	6.759	-4.774
Right_caudalanteriorcingulate	0.205	-5.772	2.034	-5.359	1.756	7.397	-4.275	-4.543	4.984	-6.074
Right_caudalmiddlefrontal	-2.909	-9.392	2.369	-6.830	5.016	11.845	-3.226	0.824	10.842	-6.235
Right_cuneus	-2.910	-7.348	0.712	-4.571	3.172	7.147	-5.996	-1.806	4.904	-5.439
Right_entorhinal	-3.270	-3.041	1.826	-0.552	4.768	4.362	1.101	6.120	4.338	-1.473
Right_fusiform	-3.094	-10.050	1.753	-5.670	4.152	11.127	-7.151	-1.864	7.951	-8.211
Right_inferiorparietal	-3.785	-10.603	3.124	-6.025	5.980	11.369	-5.175	0.578	10.417	-7.110
Right_inferiortemporal	-4.026	-10.372	0.910	-5.791	4.208	9.758	-5.806	0.306	9.246	-5.308
Right_isthmuscingulate	-1.716	-6.988	1.989	-4.614	3.234	8.021	-4.094	-1.292	5.926	-5.671
Right_lateraloccipital	-2.726	-8.887	3.057	-5.201	4.984	10.111	-4.647	-0.312	8.364	-6.861
Right_lateralorbitofrontal	-0.521	-7.478	2.153	-6.373	2.471	8.430	-4.268	-3.383	6.281	-5.879
Right_lingual	-2.550	-8.668	1.044	-4.849	2.939	7.713	-6.491	-1.679	5.869	-5.351
Right_medialorbitofrontal	-1.249	-8.210	1.638	-6.080	2.578	9.666	-5.347	-3.260	7.394	-6.822
Right_middletemporal	-4.412	-9.259	1.135	-4.345	4.726	9.080	-5.688	1.054	7.864	-5.572
Right_parahippocampal	-2.086	-4.609	0.427	-3.027	2.281	4.671	-4.066	-1.347	2.763	-3.954
Right_paracentral	-3.145	-9.399	3.598	-5.677	6.008	11.887	-3.864	0.805	10.127	-7.581
Right_parsopercularis	-1.717	-8.831	2.104	-6.710	3.538	10.771	-3.400	-0.960	10.513	-5.745
Right_parsorbitalis	-1.037	-8.201	1.202	-7.053	2.088	8.385	-1.862	-0.434	9.602	-2.902
Right_parstriangularis	-1.170	-9.611	2.862	-7.363	3.641	11.728	-2.810	-1.004	12.313	-5.921
Right_pericalcarine	-1.440	-7.735	1.464	-6.707	2.756	7.725	-6.401	-5.228	6.318	-6.056
Right_postcentral	-2.378	-8.280	2.555	-5.563	4.451	10.223	-4.260	-0.725	7.654	-6.999
Right_posteriorcingulate	-1.720	-8.646	2.098	-5.951	3.310	8.899	-5.644	-2.628	6.878	-6.295
Right_precentral	-2.645	-8.956	2.973	-5.645	5.070	12.552	-2.600	1.270	11.848	-6.719
Right_precuneus	-3.677	-9.457	2.445	-5.861	5.640	11.798	-5.092	0.139	9.135	-7.912
Right_rostralanteriorcingulate	-0.432	-8.767	1.306	-6.842	1.509	8.891	-5.696	-4.374	7.347	-5.851
Right_rostralmiddlefrontal	-1.638	-10.311	1.876	-8.069	3.226	10.821	-4.956	-2.677	12.634	-5.895
Right_superiorfrontal	-2.977	-10.937	1.783	-8.076	4.468	12.887	-5.404	-1.471	14.091	-7.068
Right_superiorparietal	-3.496	-9.008	2.976	-5.280	5.848	11.070	-4.453	0.617	9.637	-7.250
Right_superiortemporal	-4.458	-8.482	2.371	-4.468	6.332	10.995	-5.176	1.175	7.251	-8.407
Right_supramarginal	-3.375	-9.036	2.682	-5.275	5.374	10.762	-4.340	0.677	8.778	-6.905
Right_frontalpole	-1.348	-8.833	0.529	-6.266	1.602	8.496	-5.822	-3.101	6.607	-5.325
Right_temporalpole	-4.154	-5.226	2.175	-1.769	5.770	6.517	-1.838	4.195	5.966	-3.999
Right_transversetemporal	-1.372	-5.950	0.647	-4.913	1.906	6.482	-2.500	-0.772	6.761	-3.008
Right_insula	-3.106	-9.001	1.197	-5.418	3.734	9.038	-5.995	-1.204	7.212	-6.006
Left_bankssts	0.243	-5.722	4.371	-5.805	4.172	9.432	-1.464	-1.940	6.593	-7.022
Left_caudalanteriorcingulate	0.130	-5.338	1.913	-4.987	1.677	6.975	-4.042	-4.186	3.922	-6.168
Left_caudalmiddlefrontal	-1.201	-8.878	3.582	-6.826	4.362	12.006	-1.715	0.060	12.063	-6.148
Left_cuneus	-1.318	-6.380	1.703	-5.554	2.963	7.472	-4.626	-3.331	4.817	-5.985
Left_entorhinal	1.577	-1.496	2.706	-2.961	1.646	3.681	2.460	0.655	3.488	-1.486
Left_fusiform	0.488	-6.468	3.593	-6.256	2.882	10.444	-1.968	-2.652	7.664	-7.226
Left_inferiorparietal	-0.817	-8.415	4.289	-6.120	4.445	10.809	-2.340	-0.891	10.003	-6.694

Left_inferiortemporal	0.278	-8.129	3.017	-6.369	2.324	8.883	-3.727	-3.324	7.807	-5.771
Left_isthmuscingulate	-0.926	-6.961	1.012	-5.532	1.769	7.824	-4.497	-3.117	6.291	-5.235
Left_lateraloccipital	-1.024	-7.801	3.180	-5.580	3.585	9.815	-3.855	-1.888	7.054	-7.059
Left_lateralorbitofrontal	-0.001	-8.050	2.109	-6.546	1.856	8.301	-4.073	-3.467	7.215	-5.156
Left_lingual	-0.270	-6.005	1.660	-4.894	1.713	6.837	-5.138	-4.266	3.909	-6.026
Left_medialorbitofrontal	-0.516	-8.764	2.230	-6.453	2.327	9.684	-4.156	-2.768	8.935	-5.790
Left_middletemporal	0.184	-6.877	3.770	-6.510	3.424	10.468	-1.239	-1.522	8.760	-6.301
Left_parahippocampal	-0.400	-3.399	1.522	-3.419	2.044	4.562	-1.572	-1.323	3.080	-3.397
Left_paracentral	-0.823	-6.556	4.202	-5.630	4.885	11.480	-0.107	1.103	9.993	-6.383
Left_parsopercularis	-1.508	-9.111	2.309	-6.743	3.422	11.776	-3.159	-0.851	10.297	-6.348
Left_parsorbitalis	-0.097	-8.344	2.684	-7.265	2.609	9.252	-3.000	-2.707	8.941	-5.218
Left_parstriangularis	-0.836	-8.609	3.199	-6.721	3.657	9.687	-3.333	-1.896	9.112	-5.888
Left_pericalcarine	-0.817	-7.220	0.908	-6.262	1.566	6.445	-7.144	-6.127	3.816	-5.595
Left_postcentral	-1.220	-8.101	3.176	-6.505	4.110	10.194	-3.295	-1.548	8.451	-6.561
Left_posteriorcingulate	-0.494	-8.522	1.973	-6.121	2.030	9.467	-5.368	-3.653	7.262	-6.630
Left_precentral	-1.741	-8.104	3.170	-6.441	4.769	11.743	-1.760	0.678	11.459	-6.115
Left_precuneus	-1.003	-7.205	3.934	-6.183	4.820	10.779	-1.393	-0.008	9.094	-6.525
Left_rostralanteriorcingulate	-1.288	-7.938	1.669	-7.307	2.940	9.147	-4.506	-3.354	7.345	-5.976
Left_rostralmiddlefrontal	-0.695	-9.899	2.463	-8.104	2.902	11.462	-4.361	-3.218	11.625	-6.513
Left_superiorfrontal	-1.072	-10.037	2.763	-8.230	3.605	12.969	-3.758	-2.269	14.284	-6.963
Left_superiorparietal	-0.859	-6.630	4.591	-5.617	5.305	10.098	-1.182	0.021	8.532	-6.617
Left_superiortemporal	0.897	-5.585	5.018	-6.273	4.185	11.526	-0.179	-1.544	8.530	-7.934
Left_supramarginal	-1.054	-8.773	4.193	-7.089	4.879	11.970	-1.513	0.023	11.158	-6.578
Left_frontalpole	-0.893	-8.152	1.675	-5.601	2.144	7.229	-3.928	-1.889	7.138	-4.018
Left_temporalpole	-0.202	-3.940	3.240	-3.916	3.590	6.261	0.501	0.881	5.349	-3.647
Left_transversetemporal	-1.173	-5.544	3.414	-5.142	4.801	7.812	-2.698	-1.502	5.045	-6.270
Left_insula	-1.712	-6.960	1.730	-5.582	3.275	8.087	-5.394	-3.367	4.955	-6.727
Left_Accumbens_area	0.047	1.321	2.354	1.326	2.381	0.635	-1.796	-2.090	-2.621	-4.762
Left_Amygdala	4.505	4.675	8.034	1.519	5.030	2.677	3.591	-2.594	-3.452	-7.630
Left_Caudate	1.443	1.100	1.629	-0.046	0.296	0.311	1.832	-0.155	-0.046	-0.569
Left_Hippocampus	16.488	13.306	20.392	1.520	5.709	2.838	27.078	4.238	0.558	-4.165
Left_Pallidum	0.979	1.648	3.530	0.709	2.263	1.310	3.904	2.156	0.581	-1.361
Left_Putamen	1.119	1.528	3.206	0.659	2.253	1.256	1.257	-0.292	-0.994	-3.207
Left_Thalamus	4.932	4.903	7.448	1.773	4.367	2.060	9.721	6.417	2.122	-0.623
Right_Accumbens_area	-2.125	0.885	2.027	2.408	3.673	0.884	-3.569	-0.354	-3.527	-5.132
Right_Amygdala	-6.875	0.988	4.440	6.364	10.104	2.723	-4.187	5.672	-3.970	-7.399
Right_Caudate	-1.409	-0.982	0.582	0.124	1.808	1.402	-1.572	0.448	0.131	-2.150
Right_Hippocampus	-24.925	-0.978	2.473	18.143	22.495	2.720	-1.564	34.791	0.118	-3.785
Right_Pallidum	-0.171	0.483	4.055	0.550	3.825	2.690	3.254	3.156	1.392	-2.517
Right_Putamen	-2.324	0.809	2.717	2.647	4.841	1.631	-0.666	2.732	-1.575	-4.757
Right_Thalamus	-6.622	0.143	2.575	5.625	8.831	2.509	4.417	14.637	4.017	0.867

Note: S1, subtype 1; S2, subtype 2; S3, subtype 3; S4, subtype 4; HC, healthy control group.

Supplementary Table 4. Sequence of biomarkers for each trajectory via SuStaIn.

Order	Trajectory 1		Trajectory 2		Trajectory 3	
	Discovery data	Validation data	Discovery data	Validation data	Discovery data	Validation data
1	L_hippocampus	L_hippocampus	R_hippocampus	R_hippocampus	R_parstriangularis	R_precentral
2	L_thalamus	L_thalamus	R_thalamus	R_thalamus	R_parsopercularis	L_caudalmiddlefrontal
3	R_thalamus	R_hippocampus	L_thalamus	L_thalamus	R_caudalmiddlefrontal	R_caudalmiddlefrontal
4	R_pallidum	R_thalamus	L_hippocampus	L_parahippocampal	R_superiorfrontal	R_superiorfrontal
5	L_entorhinal	L_paracentral	R_pallidum	L_transversetemporal	L_superiorfrontal	L_superiorfrontal
6	L_temporalpole	L_precuneus	L_paracentral	L_temporalpole	L_caudalmiddlefrontal	L_precentral
7	L_putamen	L_caudalmiddlefrontal	L_putamen	L_hippocampus	L_paracentral	R_paracentral
8	L_paracentral	L_precentral	R_precentral	L_entorhinal	L_precentral	R_hippocampus
9	L_caudalmiddlefrontal	R_caudalmiddlefrontal	R_caudalmiddlefrontal	R_parstriangularis	R_precentral	L_paracentral
10	L_precentral	R_precuneus	L_precuneus	L_fusiform	R_precuneus	R_parstriangularis
11	L_precuneus	R_superiorfrontal	L_caudalmiddlefrontal	R_parsopercularis	R_paracentral	L_precuneus
12	L_fusiform	L_superiorfrontal	R_precuneus	R_precuneus	L_precuneus	R_precuneus
13	R_precentral	R_parsopercularis	L_precentral	L_precuneus	L_fusiform	R_thalamus
14	L_parahippocampal	R_parstriangularis	R_paracentral	L_caudalmiddlefrontal	L_transversetemporal	R_parsopercularis
15	R_caudalmiddlefrontal	R_precentral	L_entorhinal	R_superiorfrontal	L_temporalpole	L_thalamus
16	L_superiorfrontal	L_temporalpole	R_parstriangularis	L_superiorfrontal	L_entorhinal	L_hippocampus
17	R_parsopercularis	L_fusiform	L_temporalpole	R_caudalmiddlefrontal	R_thalamus	L_fusiform
18	R_precuneus	L_entorhinal	R_superiorfrontal	R_paracentral	R_hippocampus	R_pallidum
19	L_transversetemporal	R_pallidum	R_parsopercularis	L_paracentral	L_parahippocampal	L_putamen
20	R_paracentral	L_putamen	L_fusiform	L_precentral	L_thalamus	L_transversetemporal
21	R_superiorfrontal	R_paracentral	L_superiorfrontal	L_putamen	L_hippocampus	L_parahippocampal
22	R_parstriangularis	L_parahippocampal	L_parahippocampal	R_precentral	R_pallidum	L_entorhinal
23	R_hippocampus	L_transversetemporal	L_transversetemporal	R_pallidum	L_putamen	L_temporalpole

Supplementary Table 5. Consistency of individual subtype label for SuStaIn model based on 23 ROI features and 13 ROI features.

		Individual Subtype (Model_ROI13)			
		Subtype 1	Subtype 2	Subtype 3	Subtype 4
Individual Subtype (Model_ROI23)	Subtype 1	**85**	0	0	0
	Subtype 2	1	**110**	2	0
	Subtype 3	3	4	**31**	3
	Subtype 4	1	3	1	**52**

Supplementary Table 6. Consistency of individual subtype label for SuStaIn model generalized to unseen data and original subtype.

		Subtype assignment (Model generalized to unseen data)			
		Subtype 1	Subtype 2	Subtype 3	Subtype 4
Subtype assignment (original assignments)	Subtype 1	**51**	0	0	1
	Subtype 2	0	**54**	0	0
	Subtype 3	0	1	**18**	0
	Subtype 4	0	0	0	**20**

Supplementary Table 7. Prediction performance of classifiers on the test data.

Classifier Model	Kernel	Feature	Accuracy	Sensitivity	Specificity	Youden Index
Classifier A Reference	polynomial	C	67.59%	24.49%	89.58%	0.141
Classifier B1 (Subtype 1)	polynomial	C	71.15%	50.00%	80.56%	0.306
Classifier B2 (Subtype 2)	polynomial	C	70.37%	26.67%	87.18%	0.139
Classifier B3 (Subtype 3)	RBF	C&M	89.47%	85.71%	91.67%	0.774
Classifier B4 (Subtype 4)	polynomial	C	60.00%	63.63%	55.56%	0.192
Classifier B1-4 (Total)	-	-	71.72%	47.87%	81.03%	0.289

Note: C: Clinical feature set including age, sex, age of onset, illness duration, seizure lateralization, MRI HS or not, handness, history of hypoxia at birth, history of head trauma, history of febrile seizures, history of encephalitis meningitis, history of positive family, aura, seizure frequency, seizure type (FS/FBTCS), medications and pathology wave. M: MRI derived regional measures (ROI list in Supplementary Table S1).

Supplementary Table 8. Regions of interest (ROIs) included for SuStaIn modeling.

The following 23 ROI features are used for initial SuStaIn modeling		
Left Hippocampus	Right Hippocampus	Right Pallidum
Left Putamen	Left Thalamus	Right Thalamus
Left Caudal Middle frontal gyrus	Right Caudal Middle frontal gyrus	Left Entorhinal gyrus
Left Fusiform gyrus	Left Paracentral gyrus	Right Paracentral gyrus
Left Parahippocampal gyrus	Right Pars opercularis	Right Pars triangularis
Left Precentral gyrus	Right Precentral gyrus	Left Precuneus
Right Precuneus	Left Superior frontal gyrus	Right Superior frontal gyrus
Left Temporal pole	Left Transverse temporal gyrus	
The following 13 ROI features (merged from above 23 ROIs) are used for validation		
Left Hippocampus	Right Hippocampus	Right Pallidum
Left Putamen	Left Thalamus	Right Thalamus
Right Frontal lobe	Right Sensorimotor cortex	Right Parietal lobe
Left Frontal lobe	Left Sensorimotor cortex	Left Parietal lobe
Left Temporal lobe		

Supplementary Table 9. Results of linear/logistic regress model with HS as an independent variable

Y variable	ANOVA & chi-square test		X variable (HS effect)		
	F & χ^2	p*	β	t & Wals	P*
Age of onset	12.20	0.001	-4.48	-3.49	0.001
Illness duration	3.76	0.054	2.24	1.94	0.054
Medication outcome	2.89	0.089	-0.85	2.93	0.087
Surgery outcome	5.99	0.014	-1.13	5.94	0.014

* Two-sided.

Supplementary Table 10. Results of linear/logistic regress model with SuStaIn subtype as an independent variable

Y variable	ANOVA & chi-square test		X variable (SuStaIn subtype effect)		
	F & χ^2	p*	β	t & Wals	p*
Age of onset	18.85	<0.001	5.17	4.34	<0.001
Age of onset #	12.51	<0.001	4.33	3.51	0.001
Illness duration	13.73	<0.001	-3.80	-3.71	<0.001
Illness duration #	6.88	0.001	-4.00	-3.15	0.002
Medication outcome	8.43	0.004	1.50	8.47	0.004
Medication outcome #	8.58	0.014	1.39	5.64	0.018
Surgery outcome	4.42	0.036	-1.03	4.42	0.036
Surgery outcome #	8.34	0.015	-0.79	2.37	0.123

# represents a linear/logistic regress model with HS as an additional covariate variable.

* Two-sided.

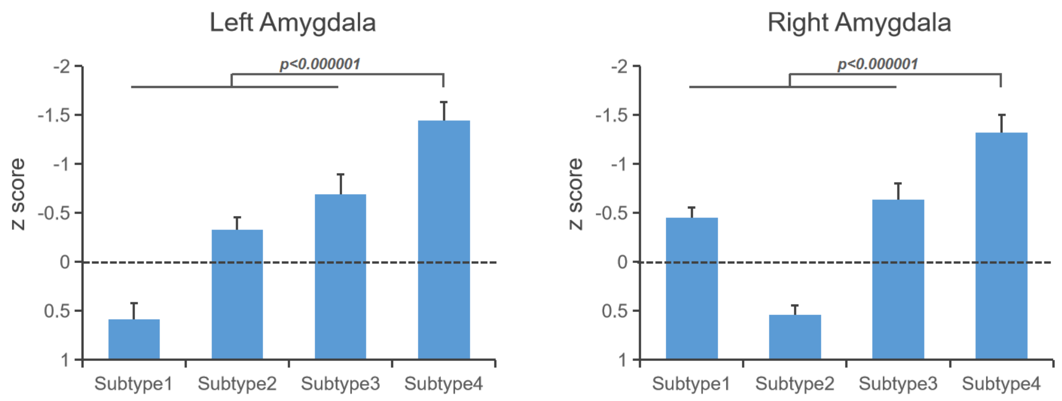

Supplementary Figure 1. ROI-wise z score of bilateral amygdala volume. Note that a negative z-score represents a larger gray matter volume relative to the healthy control group. The dashed line indicates the average of the healthy control population (i.e. z=0). The volumes of bilateral amygdala are larger in subtype 4 compared to other subtypes. Data are presented as mean values +/- SEM. n=85, 113, 41, and 57 biologically independent samples in the subtype 1, subtype 2, subtype 3 and subtype 4.

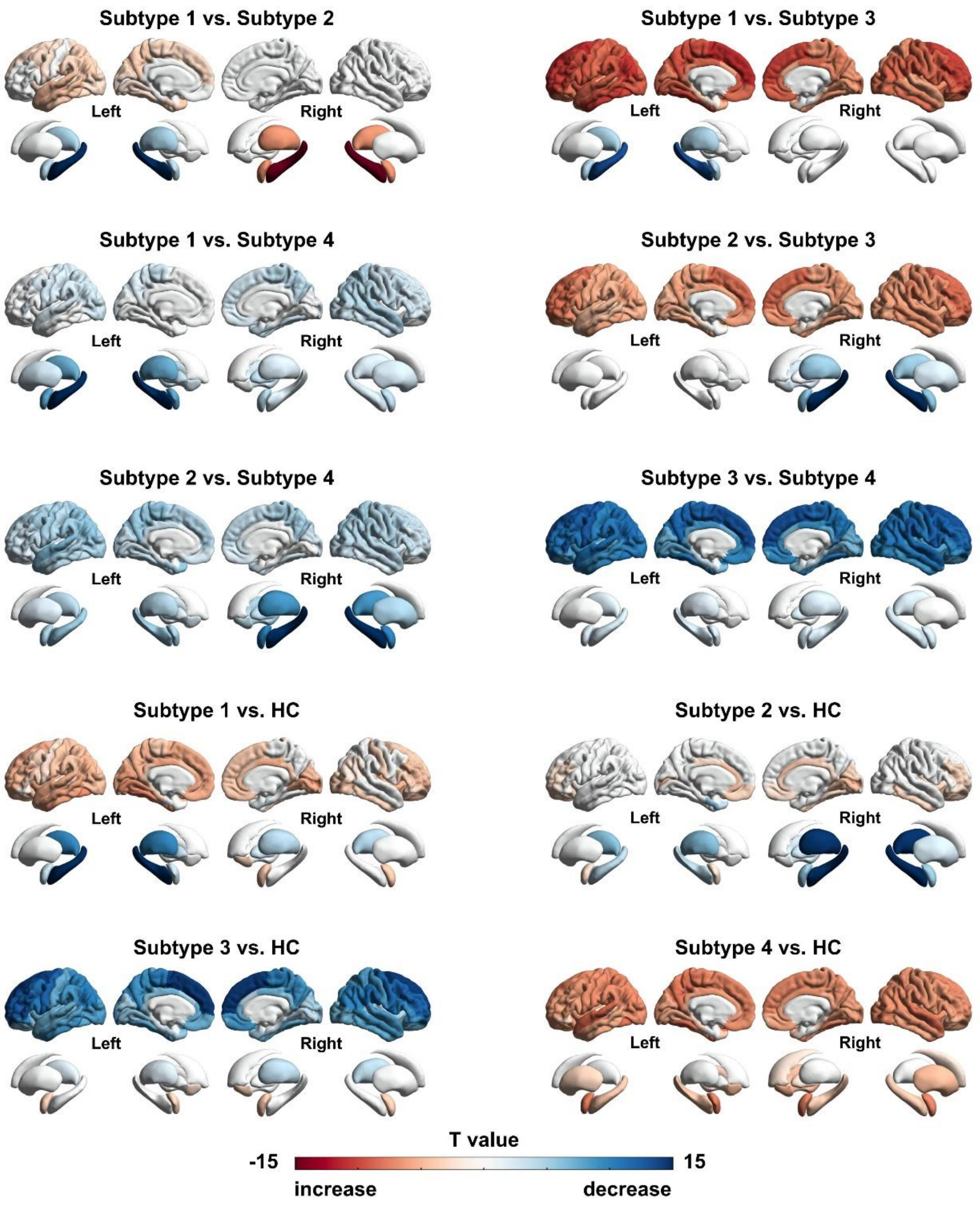

Supplementary Figure 2. Comparisons of ROI-wise z score between any two subtypes in addition to between subtypes and healthy control group (HC). Color bar indicates T value of two sample t-test with FDR correction. A positive T value (blue) represents a reduction in terms of gray matter cortical thickness or subcortical volume.

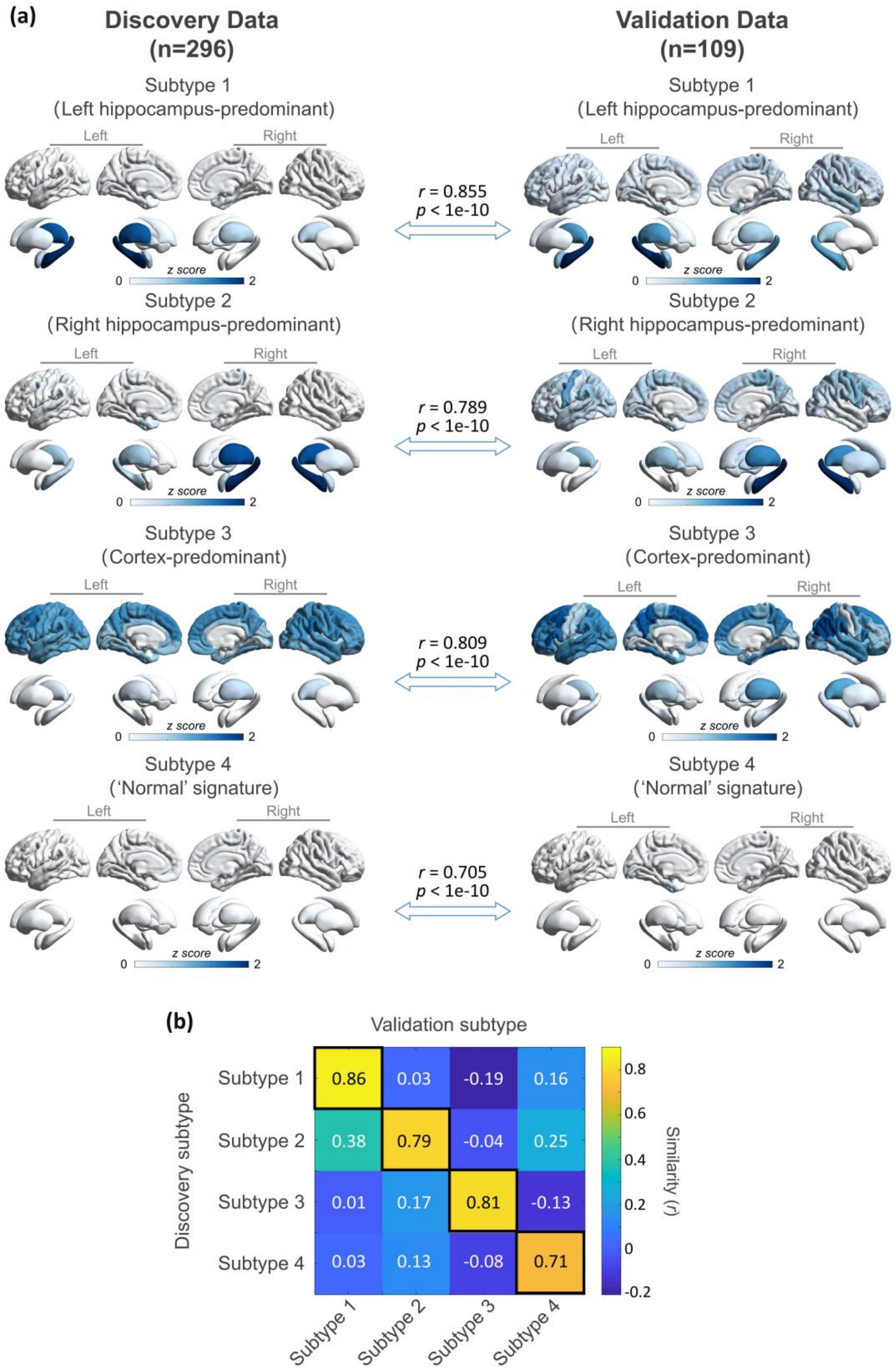

Supplementary Figure 3. Four distinct signatures of brain atrophy patterning were replicated in the validation dataset. **(a)** Four distinct neuroanatomical signatures of brain atrophy patterning in people with temporal lobe epilepsy are shown in discovery dataset and validation dataset separately. **(b)** Pearson correlation coefficient is used to evaluate the consistency of z score map between discovery dataset and validation dataset.

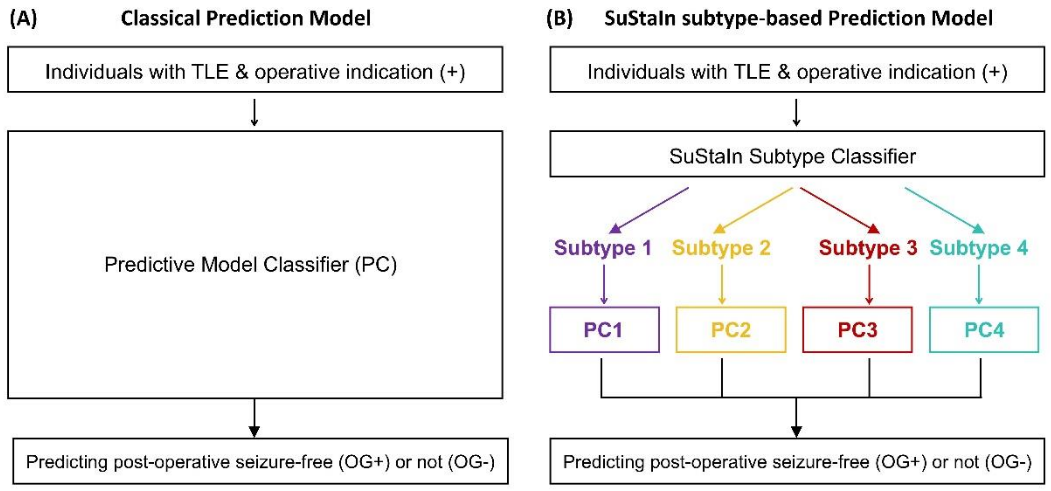

Supplementary Figure 4. Machine learning prediction procedures through a framework under SuStaIn subtype. (A) A classical prediction model without any SuStaIn subtype information. (B) SuStaIn subtype-based prediction model with specific sub-classifier corresponding to each SuStaIn subtype.

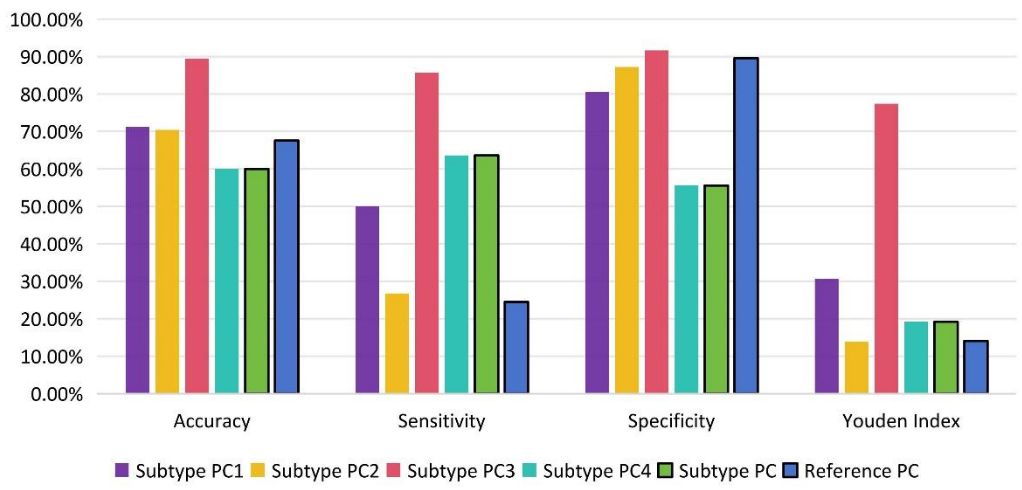

Supplementary Figure 5. Prediction performance of Machine learning classifier on identifying the subject who achieves seizure freedom or not after surgery. Subtype PC1-4: specific predictive model sub-classifier corresponding to SuStaIn subtype 1-4; Subtype PC: an overall classifier cluster of Subtype PC1-4; Reference PC: predictive classifier without any SuStaIn subtype information.

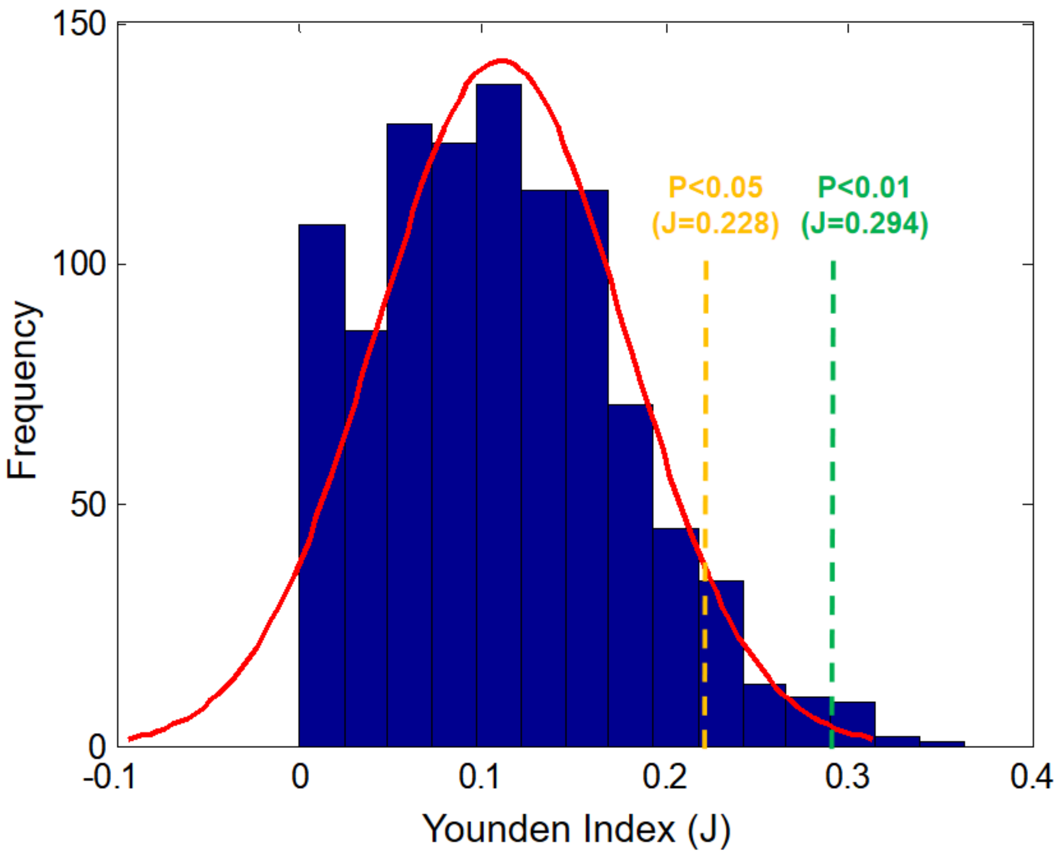

Supplementary Figure 6. Null hypothesis distributions are shown for prediction performance (Younden Index J). One-side P<0.05 (i.e., better than 95% of random predictions) is considered as a significance that rejects the null hypothesis.

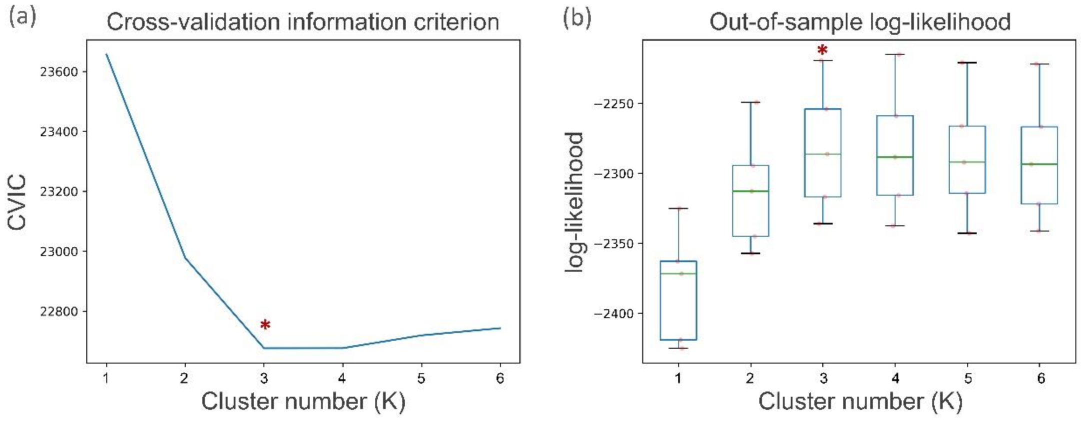

Supplementary Figure 7. Optimal number of clusters. Optimal number of clusters (i.e., trajectories) is determined using **(a)** the cross-validation information criterion (CVIC) and **(b)** the out-of-sample log-likelihood with a 5-fold cross-validation. Lower CVIC and higher log-likelihood represents better model fit.

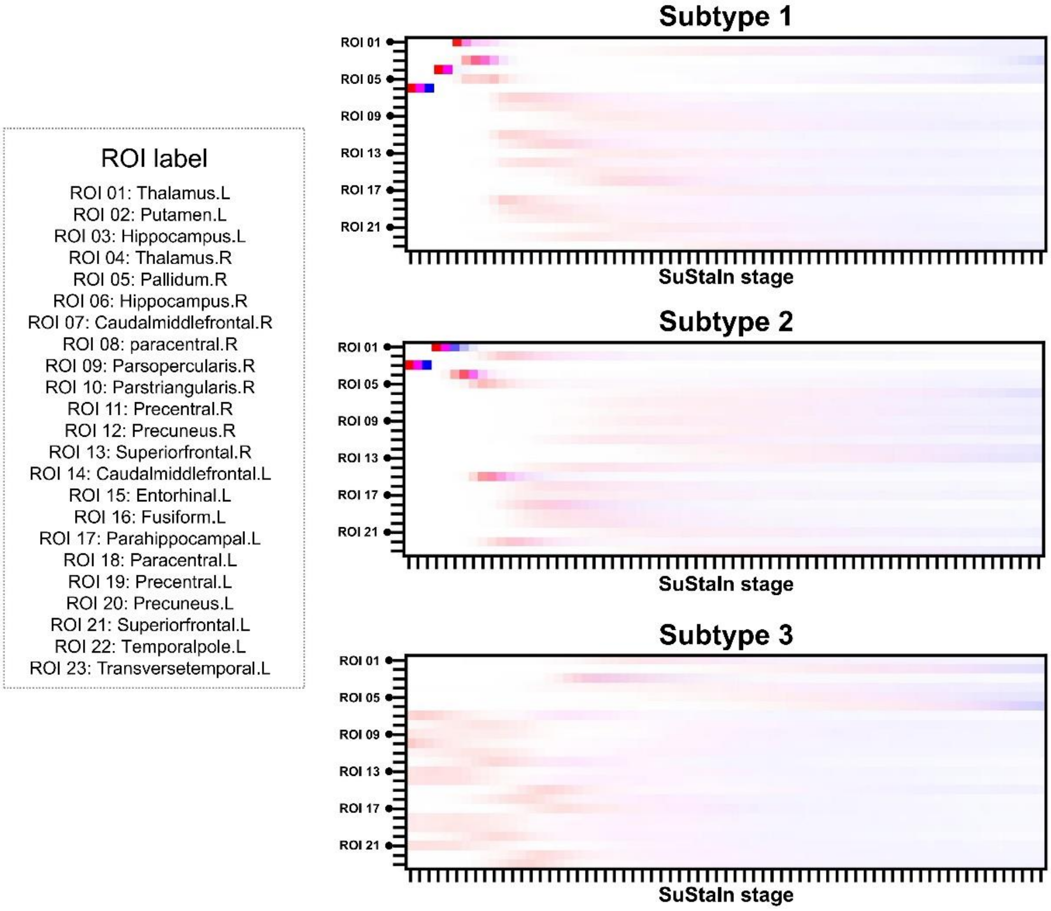

Supplementary Figure 8. Positional variance diagrams visualize the cumulative probability that each region of interested (ROI) has reached a particular z-score (1, 2 or 3) labelled by three colors. The color indicates the level of severity of gray matter reduction: red is mildly affected (z-score=1, i.e., 1 standard deviation unit from healthy control average); purple is moderately affected (z-score=2); and blue is severely affected (z-score=3).

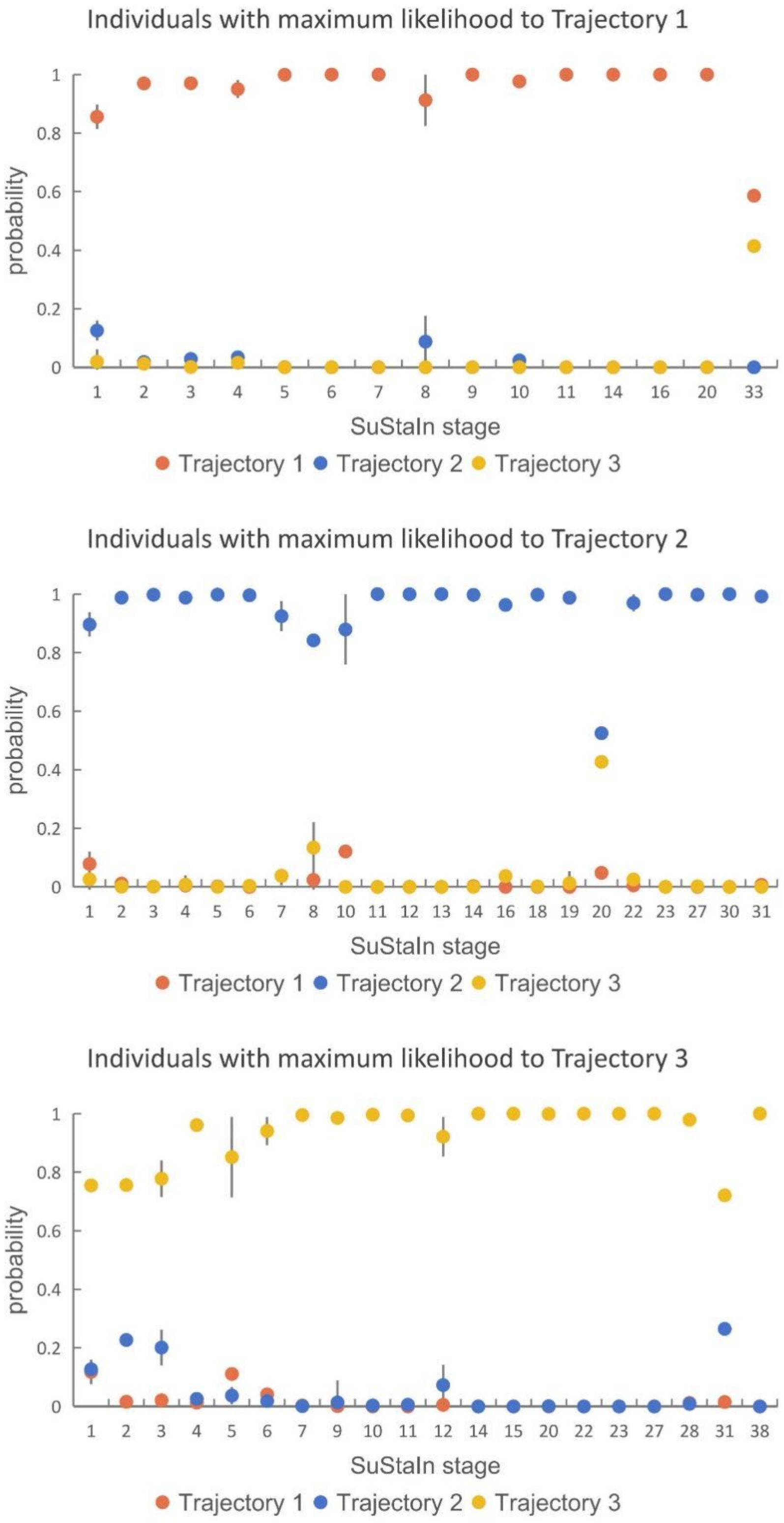

Supplementary Figure 9. Probability of maximum likelihood trajectory is high across almost all SuStaIn stages.

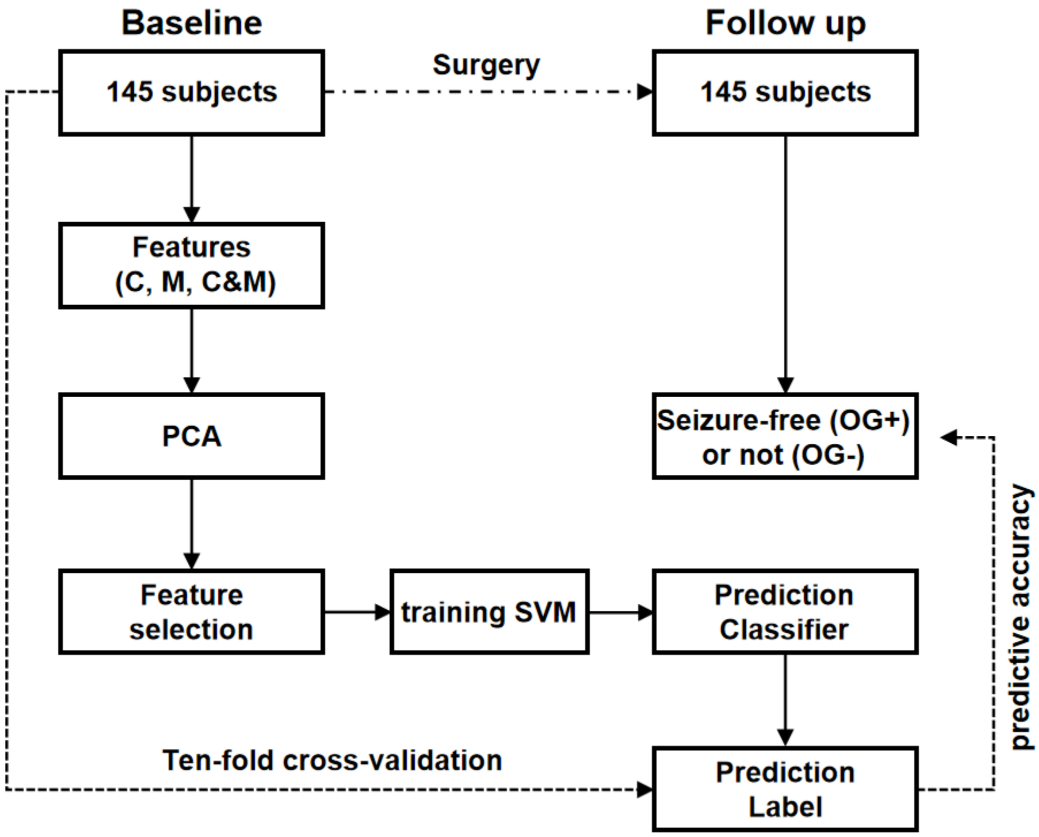

Supplementary Figure 10. A flowchart of machine learning procedures to predict treatment outcome in a sub-sample of 145 post-surgery follow-up subjects. Ten-fold cross-validation is used to obtain train data and test data. In each fold, 90% of subjects was used as a training set, and the left-out 10% subjects were used as a test set. In training set, the classifier features include the baseline clinical variables (C), MRI variables (M), or both (C&M). Principal component analysis (PCA) is used to reduce feature dimension. The first N principal components (PCs), which explained beyond 95% of the variance of all features, are used to train a support vector machine (SVM) classifier to classify the subject who achieves seizure freedom (OG+) or not (OG-) after surgery. The test set patient's label is predicted based on the trained SVM classifier. C: Clinical feature set including age, sex, age of onset, illness duration, seizure lateralization, MRI HS or not, handness, history of hypoxia at birth, history of head trauma, history of febrile seizures, history of encephalitis meningitis, history of positive family, aura, seizure frequency, seizure type (FS/FBTCS), medications and pathology wave. M: MRI derived regional measures (ROI list in Supplementary Table S7).

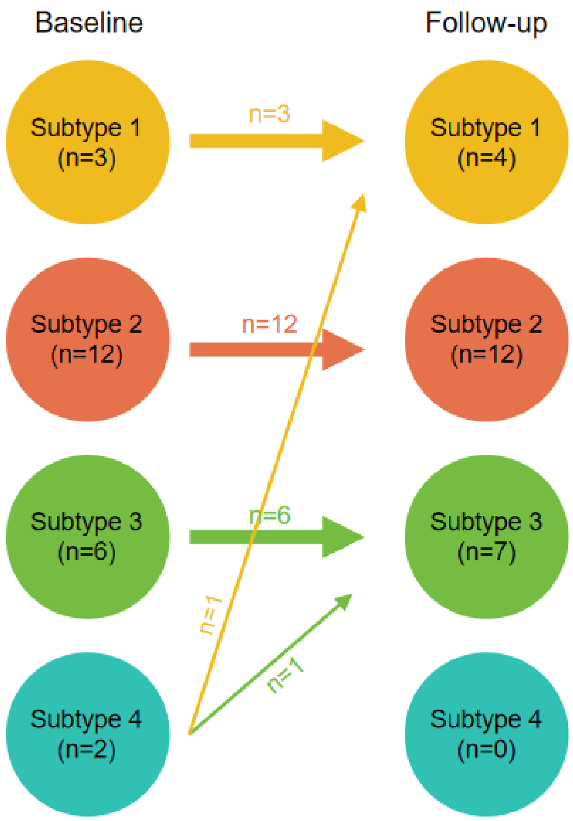

Supplementary Figure 11. Subtype labels remain consistent for almost all patients at baseline and follow-up.

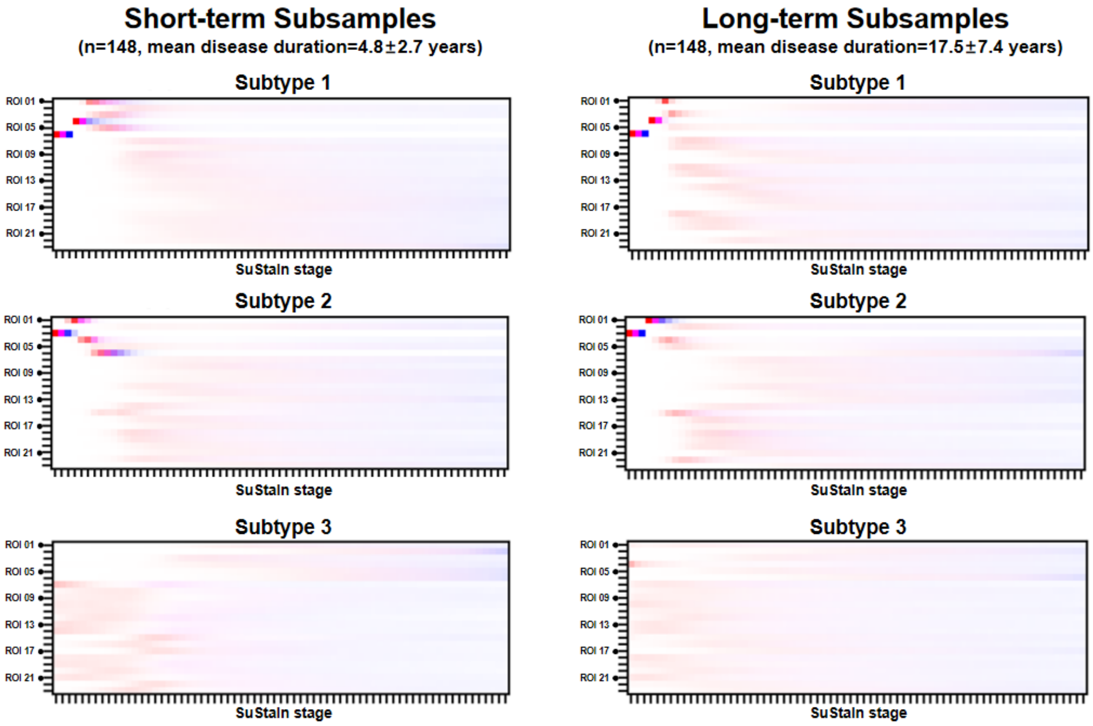

Supplementary Figure 12. Spatiotemporal trajectories of brain atrophy (visualized by positional variance diagrams) in a short-term subsample (n=148, mean disease duration=4.8±2.7 years) and a long-term subsample (n=148, mean disease duration=17.5±7.4 years), separately. Positional variance diagrams visualize the cumulative probability that each region of interested (ROI) has reached a particular z-score (1, 2 or 3) labelled by three colors. The color indicates the level of severity of gray matter reduction: red is mildly affected (z-score=1, i.e., 1 standard deviation unit from healthy control average); purple is moderately affected (z-score=2); and blue is severely affected (z-score=3).

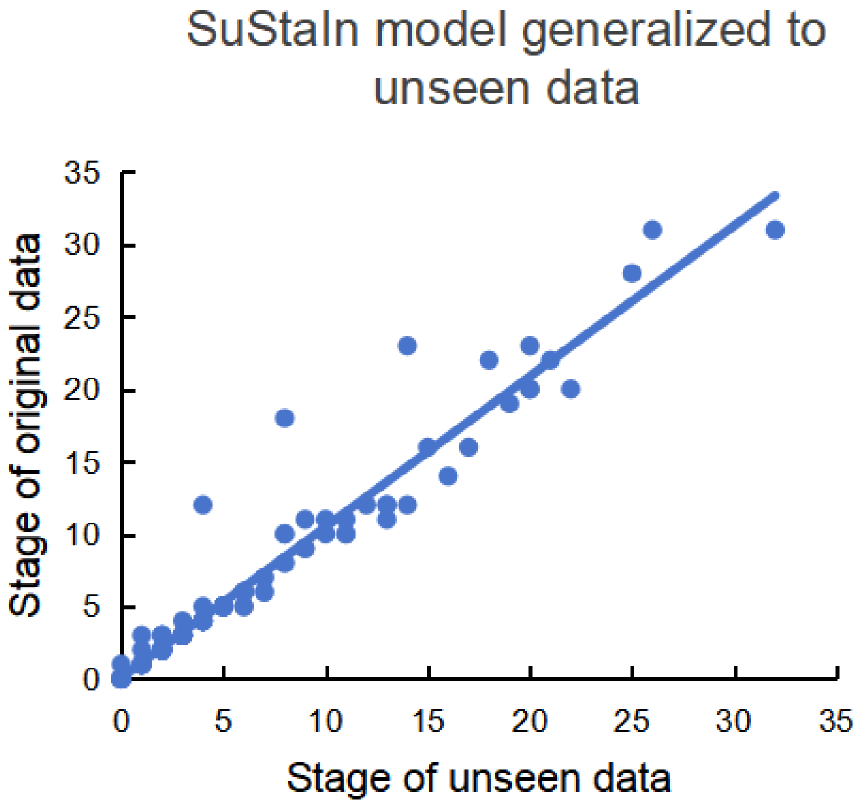

Supplementary Figure 13. Consistency of individual staging between stages of unseen data and original result ($r=0.986$, $p<0.001$, Spearman correlation test).

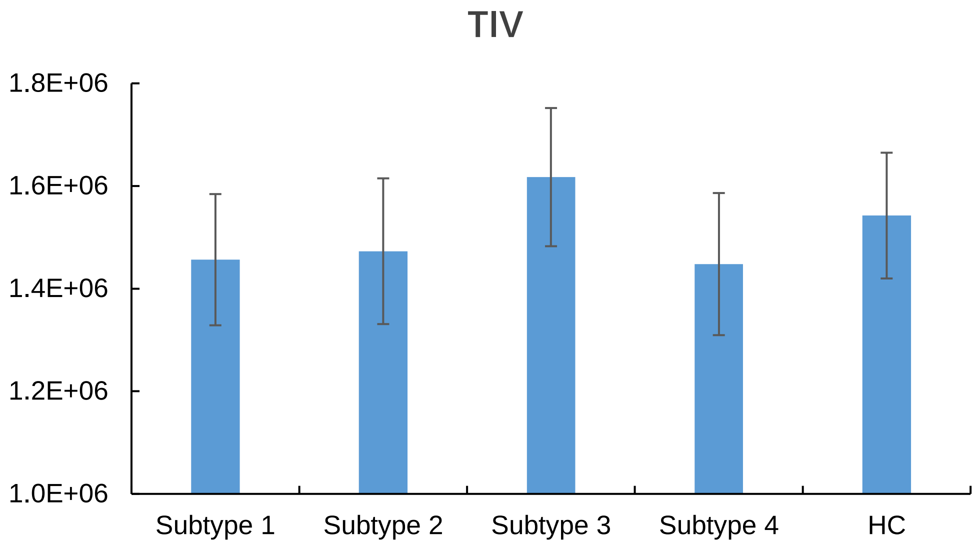

Supplementary Figure 14. Comparisons of total intracranial volume (TIV) among subtype 1, 2, 3, 4 and healthy control (HC).
