# Supplementary material for: Identification of four biotypes in temporal lobe epilepsy via machine learning on brain images
Source: Nat Commun. 2024 Mar 12;15:2221. doi: 10.1038/s41467-024-46629-6 (PMC10933450; doi:10.1038/s41467-024-46629-6)
Supplement: Supplementary file 3 — Reporting Summary [file 41467_2024_46629_MOESM3_ESM.pdf]

nature portfolio

Corresponding author(s): Yuchao Jiang, Cheng Luo, Dong Zhou, Dongmei An

Last updated by author(s): Feb 26, 2024

Reporting Summary

Nature Portfolio wishes to improve the reproducibility of the work that we publish. This form provides structure for consistency and transparency in reporting. For further information on Nature Portfolio policies, see our Editorial Policies and the Editorial Policy Checklist.

Statistics

For all statistical analyses, confirm that the following items are present in the figure legend, table legend, main text, or Methods section.

n/a	Confirmed	
☐	☒	The exact sample size (n) for each experimental group/condition, given as a discrete number and unit of measurement
☐	☒	A statement on whether measurements were taken from distinct samples or whether the same sample was measured repeatedly
☐	☒	The statistical test(s) used AND whether they are one- or two-sided<br>*Only common tests should be described solely by name; describe more complex techniques in the Methods section.*
☐	☒	A description of all covariates tested
☐	☒	A description of any assumptions or corrections, such as tests of normality and adjustment for multiple comparisons
☐	☒	A full description of the statistical parameters including central tendency (e.g. means) or other basic estimates (e.g. regression coefficient) AND variation (e.g. standard deviation) or associated estimates of uncertainty (e.g. confidence intervals)
☐	☒	For null hypothesis testing, the test statistic (e.g. F, t, r) with confidence intervals, effect sizes, degrees of freedom and P value noted<br>*Give P values as exact values whenever suitable.*
☐	☒	For Bayesian analysis, information on the choice of priors and Markov chain Monte Carlo settings
☒	☐	For hierarchical and complex designs, identification of the appropriate level for tests and full reporting of outcomes
☐	☒	Estimates of effect sizes (e.g. Cohen's d, Pearson's r), indicating how they were calculated

Our web collection on statistics for biologists contains articles on many of the points above.

Software and code

Policy information about availability of computer code

Data collection	The primary sample consisted of 296 individuals with TLE (139 females, age=27.2±8.7 years) and 81 healthy subjects (39 females, age=26.4 ±6.7 years), recruited from January 2014 to August 2022 at West China Hospital. The inclusion criteria included that 1) patients were diagnosed with TLE according to the ILAE criteria; 2) normal MRI or with unilateral hippocampal sclerosis (HS) evidence in keeping with electroencephalo-graph (EEG) findings; 3) no evidence of bilateral HS or of a secondary extrahippocampal lesion that may contribute to seizures. The exclusion criteria were as follows: 1) patients with other neurological disorder, psychiatric disorder or serious systemic disease; 2) with alcohol or other substances abuse; 3) with other structural lesions except HS according to ILAE classification confirmed by postoperative histopathological examination. Patients underwent comprehensive multidisciplinary evaluations, combining the ictal semiology, ictal and interictal EEG, MRI and PET/CT if available, to localize the seizure focus. In addition, a validation sample consisted of 109 patients (61 females, age=33.1±10.4 years) diagnosed with TLE from three local hospitals(First affiliated hospital of Zhejiang University, N=73; Fourth affiliated hospital of Zhejiang University, N=21; First affiliated hospital of Hainan Medical University, N=15) . No software was used for data collection.
Data analysis	T1-weighted structural brain MRI scans were acquired. T1-weighted images were processed using FreeSurfer (version 6.0, http:// surfer.nmr.mgh. harvard.edu/). Gray matter volumetric (GMV) measures values were estimated for 12 subcortical regions of interest (ROIs) including bilateral hippocampus, amygdala, caudate, nucleus accumbens, pallidum, putamen and thalamus. Cortical thickness (CT) measures were estimated for 64 cortical ROIs based on the DK atlas. We employed a novel AI approach (i.e., SuStaIn) to identify distinct patterns of spatiotemporal progression of brain atrophy from cross-sectional only MRI data and cluster individuals into groups (subtypes).

For manuscripts utilizing custom algorithms or software that are central to the research but not yet described in published literature, software must be made available to editors and reviewers. We strongly encourage code deposition in a community repository (e.g. GitHub). See the Nature Portfolio guidelines for submitting code & software for further information.

Data

Policy information about availability of data

All manuscripts must include a data availability statement. This statement should provide the following information, where applicable:

- Accession codes, unique identifiers, or web links for publicly available datasets
- A description of any restrictions on data availability
- For clinical datasets or third party data, please ensure that the statement adheres to our policy

The raw image and clinical data are protected and are not available due to data privacy laws. Requests for raw data can be made to the corresponding author and will be promptly reviewed by the local ethics committee to verify whether the request is subject to any intellectual property or confidentiality obligations. The processed data and statistical results are provided in the Supplementary Information/Source Data file. Source data are provided with this paper.

Research involving human participants, their data, or biological material

Policy information about studies with human participants or human data. See also policy information about sex, gender (identity/presentation), and sexual orientation and race, ethnicity and racism.

Reporting on sex and gender	Term 'sex' was used due to the biological attribution. The sex was determined based on self-reporting. This study analyzed cross-sectional T1-weighted structural MRI data from a primary sample consisted of 296 individuals with TLE (139 females, age=27.2±8.7 years) and 81 healthy subjects (39 females, age=26.4±6.7 years), as well as a validation sample consisted of 109 individuals diagnosed with TLE (61 females, age=33.1±10.4 years).
Reporting on race, ethnicity, or other socially relevant groupings	All of subjects were Chinese Han and recruited from Chinese mainland.
Population characteristics	This information is provided in detail in Table 1, but for a summary: cross-sectional T1-weighted structural MRI data came from a primary sample consisted of 296 individuals with TLE (139 females, age=27.2±8.7 years) and 81 healthy subjects (39 females, age=26.4±6.7 years), as well as a validation sample consisted of 109 individuals diagnosed with TLE (61 females, age=33.1±10.4 years).
Recruitment	The primary sample consisted of 296 individuals with TLE (139 females, age=27.2±8.7 years) and 81 healthy subjects (39 females, age=26.4±6.7 years), recruited from January 2014 to August 2022 at West China Hospital.<br>Patients diagnosed with TLE were consecutively recruited if they met the following inclusion criteria. The inclusion criteria included that 1) patients were diagnosed with TLE according to the ILAE criteria; 2) normal MRI or with unilateral hippocampal sclerosis (HS) evidence in keeping with electroencephalo-graph (EEG) findings; 3) no evidence of bilateral HS or of a secondary extrahippocampal lesion that may contribute to seizures. The exclusion criteria were as follows: 1) patients with other neurological disorder, psychiatric disorder or serious systemic disease; 2) with alcohol or other substances abuse; 3) with other structural lesions except HS according to ILAE classification confirmed by postoperative histopathological examination. Informed consent was obtained from participants or their legal guardians.<br>Patients were recruited from a tertiary comprehensive epilepsy center, those patients with newly disgnosed temporal lobe epilepsy or with well-controlled seizures by antiseizure medications were less prevalent in the study cohort. This may be one of biases. The subjective willingness of patients to participate in the experiment may be another possible bias. Since the patients were mainly Han Chinese, race and culture may also be potential biases.
Ethics oversight	This study was registered in the Chinese Clinical Trials Registry (number: ChiCTR2200062562). This study was approved by the local ethics committee of West China Hospital (ethics number: 2022-906).

Note that full information on the approval of the study protocol must also be provided in the manuscript.

Field-specific reporting

Please select the one below that is the best fit for your research. If you are not sure, read the appropriate sections before making your selection.

☒ Life sciences ☐ Behavioural & social sciences ☐ Ecological, evolutionary & environmental sciences

For a reference copy of the document with all sections, see nature.com/documents/nr-reporting-summary-flat.pdf

Life sciences study design

All studies must disclose on these points even when the disclosure is negative.

Sample size	This is a data-driven study. We included all individuals with TLE who agreed to participant in this study with available raw clinical and imaging data. The comprehensive effect size/power calculations have not been comprehensively investigated for SuStaIn. According to previous study (Vogel et al. 2021;Young et al. 2018), the required sample size is linked with feature number in SuStaIn; however, exact association is still not clear. Thus, we verified the stability of SuStaIn under different numbers of features, based on the current sample size.

Data exclusions	Individuals with poor image quality were excluded. No one was excluded because we took multiple scans of each subject to ensure data availability. In addition, participants were excluded from longitudinal analyses if they lost at follow-up.
Replication	We examined the reproducibility of the SuStaIn trajectories in another independent validation sample including 109 patients diagnosed with temporal lobe epilepsy. The three SuStaIn trajectories were successfully replicated.
Randomization	This is not relevant because we did not conduct a controlled trial.
Blinding	SuStaIn is a data-driven algorithm on patient's cross-sectional data without any prior clinical information or treatments. Thus, blinding was not relevant for experiments because we used a data-driven method.

Behavioural & social sciences study design

All studies must disclose on these points even when the disclosure is negative.

Study description	not applicable
Research sample	not applicable
Sampling strategy	not applicable
Data collection	not applicable
Timing	not applicable
Data exclusions	not applicable
Non-participation	not applicable
Randomization	not applicable

Ecological, evolutionary & environmental sciences study design

All studies must disclose on these points even when the disclosure is negative.

Study description	not applicable
Research sample	not applicable
Sampling strategy	not applicable
Data collection	not applicable
Timing and spatial scale	not applicable
Data exclusions	not applicable
Reproducibility	not applicable
Randomization	not applicable
Blinding	not applicable

Did the study involve field work? ☐ Yes ☐ No

Field work, collection and transport

Field conditions	not applicable
Location	not applicable
Access & import/export	not applicable

Disturbance	not applicable

Reporting for specific materials, systems and methods

We require information from authors about some types of materials, experimental systems and methods used in many studies. Here, indicate whether each material, system or method listed is relevant to your study. If you are not sure if a list item applies to your research, read the appropriate section before selecting a response.

Materials & experimental systems

n/a	Involved in the study
☒	☐ Antibodies
☒	☐ Eukaryotic cell lines
☒	☐ Palaeontology and archaeology
☒	☐ Animals and other organisms
☐	☒ Clinical data
☒	☐ Dual use research of concern
☒	☐ Plants

Methods

n/a	Involved in the study
☒	☐ ChIP-seq
☒	☐ Flow cytometry
☐	☒ MRI-based neuroimaging

Antibodies

Antibodies used	not applicable
Validation	not applicable

Eukaryotic cell lines

Policy information about cell lines and Sex and Gender in Research

Cell line source(s)	not applicable
Authentication	not applicable
Mycoplasma contamination	not applicable
Commonly misidentified lines (See ICLAC register)	not applicable

Palaeontology and Archaeology

Specimen provenance	not applicable
Specimen deposition	not applicable
Dating methods	not applicable

☐ Tick this box to confirm that the raw and calibrated dates are available in the paper or in Supplementary Information.

Ethics oversight	not applicable

Note that full information on the approval of the study protocol must also be provided in the manuscript.

Animals and other research organisms

Policy information about studies involving animals; ARRIVE guidelines recommended for reporting animal research, and Sex and Gender in Research

Laboratory animals	not applicable
Wild animals	not applicable
Reporting on sex	not applicable

Field-collected samples	not applicable
Ethics oversight	not applicable

Note that full information on the approval of the study protocol must also be provided in the manuscript.

Clinical data

Policy information about clinical studies

All manuscripts should comply with the ICMJEguidelines for publication of clinical research and a completedCONSORT checklist must be included with all submissions.

Clinical trial registration	ChiCTR2200062562
Study protocol	https://www.chictr.org.cn/showproj.html?proj=176800
Data collection	Information from multi-modalities such as electroencephalography（EEG） and functional Magnetic Resonance Imaging (fMRI), structural MRI and Diffusion Tensor Imaging (DTI) would be collected. The primary samples were recruited from January 2014 to August 2022 at West China Hospital.
Outcomes	Clinical assessments were assessed. An effective outcome in the medication group was defined as freedom from seizures for a duration of at least three times the longest interseizure interval before treatment or 12 months (whichever is longer), according to the criteria proposed by Kwan et al. (2011). Patients who remained seizure-free after surgery were defined to have achieved an effective outcome, following the ILAE classification.<br>Neuroimaging biomarker that was measured by MRI scanning. T1-weighted brain images were processed using FreeSurfer (version 6.0, http://surfer.nmr.mgh. harvard.edu/). Gray matter volumetric (GMV) measures values were defined as brain outcomes.

Dual use research of concern

Policy information about dual use research of concern

Hazards

Could the accidental, deliberate or reckless misuse of agents or technologies generated in the work, or the application of information presented in the manuscript, pose a threat to:

No	Yes	
☒	☐	Public health
☒	☐	National security
☒	☐	Crops and/or livestock
☒	☐	Ecosystems
☒	☐	Any other significant area

Experiments of concern

Does the work involve any of these experiments of concern:

No	Yes	
☒	☐	Demonstrate how to render a vaccine ineffective
☒	☐	Confer resistance to therapeutically useful antibiotics or antiviral agents
☒	☐	Enhance the virulence of a pathogen or render a nonpathogen virulent
☒	☐	Increase transmissibility of a pathogen
☒	☐	Alter the host range of a pathogen
☒	☐	Enable evasion of diagnostic/detection modalities
☒	☐	Enable the weaponization of a biological agent or toxin
☒	☐	Any other potentially harmful combination of experiments and agents

Plants

Seed stocks | n/a

Novel plant genotypes | n/a

Authentication | n/a

ChIP-seq

Data deposition

- [ ] Confirm that both raw and final processed data have been deposited in a public database such as GEO.
- [ ] Confirm that you have deposited or provided access to graph files (e.g. BED files) for the called peaks.

Data access links
May remain private before publication.
For "Initial submission" or "Revised version" documents, provide reviewer access links. For your "Final submission" document, provide a link to the deposited data.

Files in database submission
Provide a list of all files available in the database submission.

Genome browser session
(e.g. UCSC)
Provide a link to an anonymized genome browser session for "Initial submission" and "Revised version" documents only, to enable peer review. Write "no longer applicable" for "Final submission" documents.

Methodology

Replicates
Describe the experimental replicates, specifying number, type and replicate agreement.

Sequencing depth
Describe the sequencing depth for each experiment, providing the total number of reads, uniquely mapped reads, length of reads and whether they were paired- or single-end.

Antibodies
Describe the antibodies used for the ChIP-seq experiments; as applicable, provide supplier name, catalog number, clone name, and lot number.

Peak calling parameters
Specify the command line program and parameters used for read mapping and peak calling, including the ChIP, control and index files used.

Data quality
Describe the methods used to ensure data quality in full detail, including how many peaks are at FDR 5% and above 5-fold enrichment.

Software
Describe the software used to collect and analyze the ChIP-seq data. For custom code that has been deposited into a community repository, provide accession details.

Flow Cytometry

Plots

Confirm that:

- [ ] The axis labels state the marker and fluorochrome used (e.g. CD4-FITC).
- [ ] The axis scales are clearly visible. Include numbers along axes only for bottom left plot of group (a 'group' is an analysis of identical markers).
- [ ] All plots are contour plots with outliers or pseudocolor plots.
- [ ] A numerical value for number of cells or percentage (with statistics) is provided.

Methodology

Sample preparation
Describe the sample preparation, detailing the biological source of the cells and any tissue processing steps used.

Instrument
Identify the instrument used for data collection, specifying make and model number.

Software
Describe the software used to collect and analyze the flow cytometry data. For custom code that has been deposited into a community repository, provide accession details.

Cell population abundance	*Describe the abundance of the relevant cell populations within post-sort fractions, providing details on the purity of the samples and how it was determined.*
Gating strategy	*Describe the gating strategy used for all relevant experiments, specifying the preliminary FSC/SSC gates of the starting cell population, indicating where boundaries between "positive" and "negative" staining cell populations are defined.*

☐ Tick this box to confirm that a figure exemplifying the gating strategy is provided in the Supplementary Information.

Magnetic resonance imaging

Experimental design

Design type	This is not relevant because subjects did not need to perform experiment.
Design specifications	This is not relevant because subjects did not need to perform experiment.
Behavioral performance measures	This is not relevant because subjects did not need to perform experiment.

Acquisition

Imaging type(s)	structural
Field strength	3T
Sequence & imaging parameters	High-resolution T1-weighted images were acquired on a 3 T MRI system (Trio; Siemens) with an 8-channel head coil. Images were obtained in sagittal orientation using a spoiled gradient-recalled sequence with the main parameters: repetition time=1900ms; echo time=2.26ms; flip angle=9°; slice thickness=1mm; field of view=256×256mm2; voxel size=1.0×1.0×1.0mm3.
Area of acquisition	whole brain scan

Diffusion MRI ☐ Used ☒ Not used

Preprocessing

Preprocessing software	T1-weighted images were processed using FreeSurfer (version 6.0, http://surfer.nmr.mgh. harvard.edu/).
Normalization	Preprocessing steps including spatial registration, tissue segmentation and bias correction of intensity non-uniformities were conducted using FreeSurfer.
Normalization template	Preprocessing steps including spatial registration, tissue segmentation and bias correction of intensity non-uniformities were conducted using FreeSurfer.
Noise and artifact removal	Preprocessing steps including spatial registration, tissue segmentation and bias correction of intensity non-uniformities were conducted using FreeSurfer.
Volume censoring	Preprocessing steps including spatial registration, tissue segmentation and bias correction of intensity non-uniformities were conducted using FreeSurfer.

Statistical modeling & inference

Model type and settings	A novel data-driven approach – Subtype and Stage Inference (SuStaIn) was used to perform disease progression modeling and cluster individuals while accounting for disease progression. The SuStaIn has been described previously (Young et al 2018 Nature Communications).
Effect(s) tested	This is not relevant because this study is a structural MRI study rather than task fMRI.

Specify type of analysis: ☐ Whole brain ☒ ROI-based ☐ Both

Statistic type for inference (See Eklund et al. 2016)	This is not relevant because GMV value was extracted at ROI-level.
Correction	FDR

Models & analysis

n/a	Involved in the study
☒	☐ Functional and/or effective connectivity
☒	☐ Graph analysis
☒	☐ Multivariate modeling or predictive analysis

Functional and/or effective connectivity	*Report the measures of dependence used and the model details (e.g. Pearson correlation, partial correlation, mutual information).*
Graph analysis	*Report the dependent variable and connectivity measure, specifying weighted graph or binarized graph, subject- or group-level, and the global and/or node summaries used (e.g. clustering coefficient, efficiency, etc.).*
Multivariate modeling and predictive analysis	*Specify independent variables, features extraction and dimension reduction, model, training and evaluation metrics.*
